# Interplay of Polarity Proteins and GTPases in T-Lymphocyte Function

**DOI:** 10.1155/2012/417485

**Published:** 2012-02-22

**Authors:** Ivan Fung, Sarah M. Russell, Jane Oliaro

**Affiliations:** ^1^Cancer Immunology Program, Peter MacCallum Cancer Centre, East Melbourne, VIC 3002, Australia; ^2^Department of Pathology, The University of Melbourne, Parkville, VIC 3010, Australia; ^3^Centre for Micro-Photonics, Swinburne University, Hawthorn, VIC 3122, Australia

## Abstract

Polarity refers to the asymmetric distribution of different cellular components within a cell and is central to many cell functions. In T-cells, polarity regulates the activation, migration, and effector function of cytotoxic T-cells (CTLs) during an immune response. The regulation of asymmetric cell division by polarity proteins may also dictate CTL effector and memory differentiation following antigen presentation. Small GTPases, along with their associated polarity and adaptor proteins, are critical for mediating the polarity changes necessary for T-cell activation and function, and in turn, are regulated by guanine exchange factors (GEFS) and GTPase activating proteins (GAPS). For example, a novel GEF, dedicator of cytokinesis 8 (DOCK8) was recently identified as a regulator of immune cell function and mutations in DOCK8 have been detected in patients with severe combined immunodeficiency. Both B and T-cells from DOCK8 mutant mice form defective immunological synapses and have abnormal functions, in addition to impaired immune memory development. This paper will discuss the interplay between polarity proteins and GTPases, and their role in T-cell function.

## 1. Overview of Polarity

Polarity refers to the asymmetric distribution of surface receptors, cytoskeletal components, vesicle trafficking, and signaling proteins within a cell [1]. Many polarity components are conserved between different cell types and organisms (reviewed in [2]). Polarity is an important factor in T-cell functions, such as immunological synapse (IS) formation, migration, target cell killing, asymmetric cell division (ACD), and differentiation [3–8]. In order to establish and maintain polarity in response to dynamic cell-cell interactions and extracellular cues, a T-cell must be able to orchestrate different signals to regulate the different recruitment of many cellular components. This process is highly regulated and involves both GTPases (reviewed in [9]) and a network of polarity proteins [1].

GTPases act as molecular switches to control cellular processes. The family of Rho GTPases includes Cdc42, RhoA, and Rac1 (reviewed in [10]). GTPases have two conformational states, which are dependent on the type of guanine nucleotide bound. The active state is induced by the binding of Guanosine-5′-triphosphate (GTP), and the inactive state is induced when Guanosine diphosphate (GDP) is bound. The loading of GTP and dissociation of GDP are regulated by different proteins: guanine exchange factors (GEFs) promote the exchange of GDP for GTP, GTPase activating proteins (GAPs) catalyze the activity of GTPase activity to their downstream effectors, and the guanine nucleotide dissociation inhibitors (GDIs) block regulation (reviewed in [11]). Activated Rho GTPases regulate cytoskeleton remodeling, which in turn influences morphology, migration, and protein trafficking (reviewed in [12]). Like other members of the Rho GTPase family, Cdc42 influences a large array of cellular activities. Its downstream effectors include a large number of kinases which activate many signaling pathways [13, 14] as well as nonkinase proteins, such as neuronal Wiskott-Aldrich Syndrome protein (N-WASP) [15] which promotes actin nucleation.

The evolutionarily conserved polarity proteins are localized into different regions of a cell to act as scaffolds for the recruitment of other protein complexes (reviewed in [16]). The Scribble, Par and Crumbs polarity protein complexes are the most extensively studied. The Scribble complex consists of Scribble (Scrib), Discs large (Dlg), and Lethal giant larve (Lgl) proteins (reviewed in [17]). The Scribble and Par complexes regulate asymmetric cell division (ACD) of neuroblasts in *Drosophila* (reviewed in [18]). The Par complex, which consists of Par3, Par6, and atypical protein kinase C (aPKC), was first discovered in *C. elegans* embryos that have defective anterior-posterior partitioning [19]. The Crumbs complex consists of Crumbs, PatJ, and Pals1 (reviewed in [2]) and is important in mammalian epithelial cell polarity [20]. All of these proteins, with the exception of aPKC, consist of a variable number of binding motifs termed PSD-95/Dlg/ZO-1 (PDZ) domains [2]. The PDZ domain can interact with a number of signaling proteins; for example, Dlg1 can interact with protein tyrosine phosphatase and tensin homologue (PTEN) [21] as well as with other PDZ-containing proteins and the Par6-aPKC complex can interact with Lgl, Par3, and Pals1 (reviewed in ([1, 2]). Polarity proteins establish a network to orchestrate signals throughout the cell in response to extracellular cues. The polarity proteins can work cooperatively or antagonistically [17] to regulate cell polarity. Polarity proteins also work in conjunction with GTPases to establish and maintain cell polarity (reviewed in [22]).

## 2. Polarity in T-Cells: The Immunological Synapse

Two main classes of T-cells are produced after maturation and selection in the thymus: CD8^+^ T-cells and CD4^+^ T-cells, distinguished by their expression of either the cell surface marker, cluster of differentiation 8 (CD8) or 4 (CD4). CD8^+^ T-cells function as cytotoxic T-lymphocytes (CTL) and have the ability to kill target cells, such as virus-infected cells, by releasing pore-forming perforin and serine protease granzymes via exocytosis [23]. To carry out their immune functions, CTLs must first be activated. CTL activation involves the interaction of the T-cell receptor (TCR) with pathogen-derived peptide antigen presented by antigen presenting cells (APCs) via their major histocompatibility complex (MHC) class I molecule. An immunological synapse (IS) is formed when a TCR interacts with peptide MHC (as reviewed in [24, 25]). T-cell activation also involves an important second signal, which is provided by the interaction between the costimulatory molecules on the T-cell and APC. The importance of the co-stimulatory signal in naïve T-cell activation has been demonstrated in many *in vitro* studies (reviewed in [26]).

During IS formation, many molecules and complexes are recruited towards, or away from, the cell-cell interface. Molecules such as the TCR and microtubule organizing centre (MTOC) are recruited to the interface, while CD43, a member of sialoglycoproteins, is polarized to the distal pole, away from the interface [27]. At the interface, compartmentalization of proteins was first described by Kupfer's group, where surface molecules are clustered to regions termed supramolecular activation clusters (SMACs) [28]. In a mature IS, the central region, or cSMAC, contains the TCR, CD28, and their associate signaling molecules. The cSMAC is surrounded by an outer ring of adhesion molecules including lymphocyte function-associated antigen 1 (LFA-1), and talin, a cytoskeleton protein that links integrins to the actin cytoskeleton [29, 30]. The formation of the IS is a dynamic process. Initial antigen-independent contacts between the T-cell and the target cell involve the interactions of adhesion molecules such as CD2 with LFA-3 [31] and LFA-1 with ICAM-1 [28]. LFA-1 and ICAM-1 localize to the cSMAC and TCR-MHC complexes to the pSMAC at the initial phase of IS formation. However, in a mature IS the situation inverts and the TCR-MHC complex resides in the cSMACs, while the antigen independent interactions are at the periphery [24]. An important implication of a polarized and compartmentalized IS is the regulation of T-cell activation, by controlling TCR signaling and TCR degradation [32]. Studies have shown that the cSMAC plays a role in TCR degradation in the event of strong agonist interactions [33], and it has been proposed that signals from weaker interactions are enhanced [34]. The exact role of the synapse is still controversial ([35, 36]), however, these studies highlight the importance of the polarized and compartmentalized nature of the immunological synapse.

While the nature and function of the TCR-MHC complex has been intensively studied, the role of LFA-1 and its interaction with its ligand, ICAM-1, in the pSMAC has only recently been elucidated. LFA-1 is part of the large family of leukocyte integrins and is expressed on T and B lymphocytes. It is involved in a wide range of T-cell functions including activation upon antigen presentation, CTL-mediated killing, cell adhesion, and migration. The importance of LFA-1 in the immune system is highlighted by patients with leukocyte adhesion deficiency (LAD) who have impaired pathogen clearance and suffer repeated infections [37]. Integrin *β*2 chain (CD18)–deficient mice displayed defects in leukocyte adhesion and proliferation [38]. LFA-1 is critically involved in the initial contact of a T-cell with the APC [39]. This contact is essential for T-cell activation as it provides the stop signal for a migrating T-cell to scan the surface of the APC for peptide-MHC. The TCR-peptide-MHC interaction activates LFA-1 and increases its affinity and avidity, resulting in a stringent interaction with its ligands, such as ICAM-1 (reviewed in [40]). This stronger interaction is believed to be a stabilizer in T-cell dendritic cell (DC) interactions [41] therefore allowing sustained TCR signaling. LFA-1 is also needed for Erk1/2 signaling during antigen presentation [42]. The Erk1/2 signaling pathway promotes T-cell activation and proliferation. LFA-1 is one of the many proteins that regulate IS formation and, as discussed above, is critical for normal T-cell activation and proliferation.

**Figure 1:**
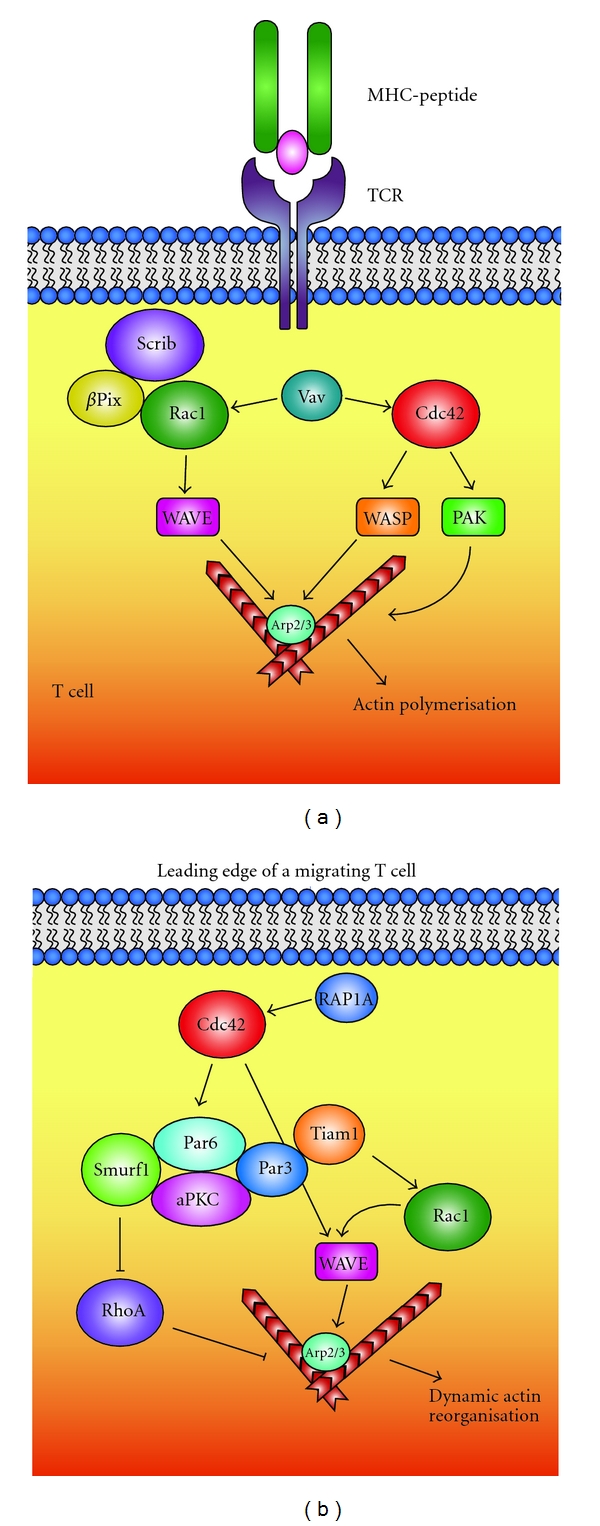
GTPases are important mechanical switches in T-lymphocyte function. (a) During antigen presentation, a T-cell undergoes dramatic changes in protein localization and morphology. The polarity protein, Scribble, is believed to be recruited to the synapse after TCR signaling and, through its potential association with *β*PIX, may recruit Rac1 and Cdc42 to close proximity to GEFs such as Vav. Activated Rac1 and Cdc42 in turn, activate downstream effectors such as WAVE, WASP, and PAK, enabling actin polymerization and thus, changes in morphology. (b) In a migrating T-cell, GTPases regulate actin polymerization to allow for cell moment. At the leading edge of the cell, Cdc42 is activated by the Ras-related protein RAP1a, which in turns activates members of the Par complex. Par3 recruits a RAC GEF, Tiam1, which in turn activates Rac1. Rac1 promotes actin reorganization, thus lamellipodium formation through proteins such as WAVE and Arp2/3. The Par complex also binds and activates the E3 ligase Smurf1. Smurf1 promotes degradation of another GTPase, RhoA, which, in its active form enables actin contractility in cells.

To carry out its highly specialized functions, the IS and its associated signaling and adhesion proteins are tightly regulated. The change in morphology that occurs when a T-cell contacts a target cell is mediated by actin cytoskeleton rearrangement. TCR signaling induces phosphorylation of myosin II [43], which causes loss of myosin filaments [44]. This allows for the depolymerization of the actin cytoskeleton in the midbody, and in the uropod, facilitating change in morphology. The Scribble complex is also believed to be involved in myosin II regulation [45]. Scribble and Dlg are transiently recruited to the cell-cell interface upon IS formation [6, 46]. TCR signaling induces dephosphorylation of pERM, which leads to relaxation of the cytoskeleton, allowing Scribble and Dlg to be recruited to the synapse. This process is mediated by cytoskeleton rearrangements that are regulated by Rho and Rac GTPases [47]. TCR signaling also leads to Vav-(a GEF for Cdc42) mediated cytoskeleton remodeling. After activation by TCR signaling, Vav activates Cdc42 and Rac1 [48, 49], which in turn activates WASP and PAK. WASP promotes actin nucleation, which generates a contracted actin network that serves as a scaffold for signaling molecule recruitment. Scribble may recruit Rac1 and Cdc42 to the IS through the p21-activated kinase [PAK]-interacting exchange factor (*β*-PIX) [50] and may bring the GTPases into close proximity to their downstream effectors and many signaling molecules (Figure 1(a)). *β*-PIX and Scribble have been shown to interact in other cell types, so this interaction may also provide a mechanism for recruitment of *β*-PIX to the IS following TCR stimulation. TCR signaling also leads to the activation of downstream transcription factors, which play a major role in regulating asymmetric cell division and differentiation, and polarity proteins may serve as an integrating platform for various signals.

## 3. Polarity in T-Cells: Asymmetric Cell Division

As discussed above, a naïve T-cell is activated after interacting with the peptide-MHC molecule on APCs, during which an IS is formed. Differentiated cells are well-characterized by the expression levels of specific cell surface markers and immune functions, as well as transcriptional events in the developmental pathway. However, there are competing hypotheses on the mechanism that gives rise to the large variety of functionally diverse subsets of T-cells [51–56]. In the “one cell, one fate” model, naïve cells are activated after receiving unique signals and give rise to a homogeneous populations of progeny cells. The generation of different subsets of cells is therefore determined by factors such as antigen availability over time and degree of maturation of the DCs [57]. The “one cell, multiple fates” model proposes that naïve cells undergo asymmetric cell division after activation and give rise to two daughter cells that are committed to different cell fates, thus generating a heterogeneous population of progenies [58]. Asymmetric cell division involves the establishment of an axis of polarity, which may be influenced by different external cues such as the microenvironment and the orientation of the mitotic spindle to the axis. Fate determinants are recruited into the two daughter cells and after division, each daughter cell inherits a different set of determinants, which set them on different paths of cell fate (reviewed in [59]). Asymmetric cell division has been observed in different cell types in mammalian cells [60] and is evolutionarily conserved across many organisms. One example is when a *Drosophila *sensory organ precursor (SOP) undergoes asymmetric cell division to produce a pIIa cell and pIIb cell. Following another round of asymmetric cell division, the pIIa daughter cell gives rise to a socket and a shaft granddaughter cell. One of the daughter cell of pIIb is programmed to die, while the other gives rise to a neuron and a sheath cell [61].

The first evidence to show that asymmetric cell division occurs in T-lymphocytes was reported by Chang et al. [5]. This study, and others since, has shown that polarity proteins, cell fate determinants such as Numb, and transcription factors, are asymmetrically distributed in T-cells during cell division [4, 5, 8]. Most interestingly, the putative “proximal” daughter cells (isolated by high expression of CD8) provided acute, but poor long-term, protection against *Listeria* infection after adoptive transfer of the daughter cells into recipient mice. In contrast, the low CD8 expressing daughter cells (putative “distal” daughters) gave long-term protection [5]. Scribble, aPKC and Par3 are all asymmetrically distributed during cell division in T-lymphocytes. However, the mechanisms of ACD, as well as how extracellular cues, such as the degree of DC maturation and the cytokine environment, can influence asymmetric cell division and ultimately cell fate, are poorly understood.

## 4. Polarity in T-Cells: Migration

Migration is particularly important in the context of T-cell activation and effector functions, as T-cells undergo a number of scanning steps before antigen recognition. When a T-cell migrates, it establishes a front-rear polarity with a leading edge and a trailing end (reviewed in [3, 62]). The leading edge of the cell, or lamella, has a high concentration of free actin filaments to generate contractile force [3], and chemokine receptors such as CCR2 and CCR5 [63] to facilitate effective homing of the lymphocyte. The posterior of the cell contains a protrusion, termed the uropod, which adheres to the substratum, allowing the lymphocyte to move forward (reviewed in [27, 45]). The MTOC, the TCR, ezrin, and adhesion molecules, such as CD43, and intercellular adhesion molecule-1 (ICAM) [64] are polarized to the uropod. GTPases and polarity proteins regulate the spatial organization of these cellular components.

The shape of a migrating T-cell is dynamic and requires continual rearrangement of the actin cytoskeleton in the lamellipodia [65]. Therefore, a T-cell must be able to remodel its cytoskeleton efficiently. GTPases are central to this process and the spatial regulation of their activity enables cell movement and controls directionality (reviewed in [66]). Rac1 and Cdc42 promote actin nucleation at the leading edge of a T-cell via WASP and Scar proteins, which induce Arp2 and Arp3 proteins to bind to actin monomers and promote nucleation. Nucleation of actin monomers catalyzes actin polymerization [1, 67]. Rac1 promotes protrusion and Cdc42 induces filopodia. Cdc42 is also essential for directing a migrating cell to extracellular cues [68]. Another important GTPase is RhoA. Activation of RhoA is required for uropod formation. ROCK protein kinase is one of the downstream effectors of RhoA [69]. ROCK signaling results in cell body contraction and rear end retraction (reviewed [1]).

Polarity proteins have been shown to interact extensively with GTPases in T-cells and other cell systems. Following chemokine stimulation, Cdc42 at the leading edge is activated by RAP1A, a Ras-related protein, which activates the Par complex [22]. Tiam1, a Rac GEF, is recruited to the leading edge by Par3 [70, 71] and then activates Rac1, which in turn induces actin nucleation and therefore lamellipodium formation. The Par6-aPKC heterodimer also binds to E3 ligase Smurf1 and activates it. Smurf1 degrades RhoA [72] and therefore reduces actin contractility, resulting in the characteristic dynamic actin polymerization and depolymerization at the leading edge (Figure 1(b)). Scribble and Dlg are found to be asymmetrically distributed in the uropod of migrating T-cells and reduced expression of Scribble and Dlg by shRNA knockdown results in the loss of the uropod and the recruitment of the uropod markers, CD44, and Ezrin. The loss of Scribble also causes abrogation of T-lymphocyte migration [6].

## 5. DOCK8: A New Player in T-Cell Polarity

Apart from the more extensively studied polarity proteins, the protein Dedicator of Cytokinesis 8 (DOCK8), was recently identified as a potential regulator of polarity in immune cells. DOCK8 is a Rho-Rac guanine exchange factor [73] and was first discovered in a screen for binding partners of the Rho GTPase, Cdc42 using a yeast two-hybrid system [74]. The DOCK8 protein has extensive homology to the Ced-5/DOCK180/Myoblast city (CDM) family of proteins [75]. The members of this family of proteins share two conserved domains, DOCK homology regions (DHR) 1 and 2. The important GEF activity is situated in the DHR-2 domain. There are eleven members in the DOCK family [74] but only DOCK180 and DOCK2 have been extensively studied. DOCK2 is required for CD28-mediated Rac activation [76], translocation of the TCR after antigen presentation [77] and lymphocyte migration [78]. DOCK2 and DOCK180 are involved in cytoskeletal remodeling [78, 79] and in regulating the activation of Rac [77, 80]. Apart from binding to Cdc42 with high affinity in the yeast two-hybrid screen, Ruusala also demonstrated that DOCK8 is localized in the lamellipodia in porcine aortic endothelial cells [74] where extensive actin cytoskeleton remodeling occurs. Therefore, one can speculate that, similar to the other members in the family, DOCK8 is involved in some aspects of actin cytoskeleton regulation. This is reinforced by the fact that DOCK8 serves as a GEF for Cdc42, which is a regulator of cell morphology, migration, and proliferation.

Interestingly, loss-of-function mutations in *DOCK8* were recently identified in patients with severe combined immunodeficiency, characterized by repeated bacterial and viral infections [81, 82]. Analysis of patient lymphocytes revealed lower numbers of CD4^+^ and CD8^+^ T-cells, impairment of T-cell proliferation upon stimulation by anti-CD3 and anti-CD28 antibodies, and a moderate decrease in interferon-*γ* (IFN-*γ*) and tumour necrosis factor-*α* (TNF-*α*) [81, 82]. However, the CD8^+^ T-cells had normal levels of cytotoxic activity as well as extravasation ability [82]. These studies demonstrated, for the first time, that DOCK8 is involved in the regulation of immune cells. DOCK8 has also been speculated to have tumour-suppressor functions, as a number of patients in the study had human papillomavirus infections and cutaneous T-cell lymphoma-leukemia [82].

Using a DOCK8 mutant mouse model, Primurus (*pri/pri*), where a point mutation changes a serine to a proline residue in the DHR-2 domain of the DOCK8 protein, Randall et al., [73] have characterized the role of DOCK8 in immune cell function. The *pri/pri* mutation is thought to break the contact between the DHR-2 domain with Cdc42, and therefore interfere with normal GTP exchange function. Analysis of the *pri/pri* mice revealed that there are defects in marginal zone B lymphocyte formation as well as in B-cell persistence in the germinal centers. The mutant B-cells are also unable to undergo affinity maturation, resulting in poor longevity in memory-mediated humoral response. The mutation also disrupts the accumulation of ICAM-1 to the pSMAC of the IS [73]. DOCK2 deficient mice also have impaired B-cell migration to lymph nodes but this phenotype is not observed in the *pri/pri* mice despite the high degree in homology between amino acid sequence between DOCK2 and DOCK8 [74]. This data suggests that DOCK8 may have a specialized role in immune cells.

The severe cutaneous viral infections typical of patients with DOCK8-deficiency in particular, suggest a role for DOCK8 in CD8^+^ T-cell function. In two separate studies using the *pri/pri* mouse model, mutation of DOCK8 significantly decreased the number of peripheral naïve CD8^+^ T-cells [83, 84]. Although phenotypically normal, the CD8^+^ T-cells show delayed proliferation in response to dendritic cells presenting antigen *in vitro* [83]. Despite this phenotype, DOCK8 deficient mice mount a relatively normal primary immune response to viral infection *in vivo*, but show significantly impaired persistence and survival of memory CD8^+^ T-cells [83, 84]. Interestingly, this defect correlated with abnormal polarization of LFA-1 and actin to the immunological synapse formed between naïve CD8^+^ T-cells and antigen-presenting dendritic cells [73] suggesting a polarity defect that results in suboptimal synapse formation. These data, and others [85, 86], suggest that the quality of the IS and the downstream signals generated are critical for the development and persistence of memory T-cells.

## 6. Conclusions

**Figure 2:**
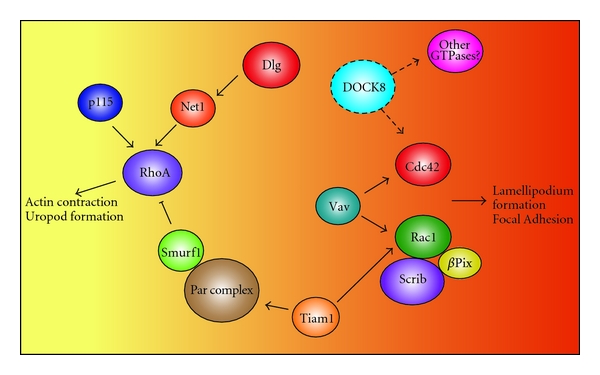
GEFs and polarity proteins are important GTPase regulators. GTPases function as switches in cells, controlling a large variety of pathways. They are tightly regulated by Guanine exchange factors (GEFS), GTPase activating proteins (GAPS) and polarity proteins. The recently discovered that GEF, DOCK8, may also be part of this large network. Evidence has shown that it interacts with Cdc42, an important GTPase in the regulation of cell morphology and motility. DOCK8 may also be a regulator of other GTPases that control different cellular functions important for T-cell function.

It is now apparent that, similar to polarity of cells of solid tissues [16], polarity of immune cells may be controlled by a dynamic and two-way interaction between polarity proteins and Rho GTPases. The molecular links between the two groups of proteins seem to be predominantly built upon physical interactions between regulators of the Rho GTPases such as the GEFS, and different components of the polarity complexes (Figure 2). As we identify the specific role of each GEF in morphological changes of immune cells, we will begin to elucidate how the polarity proteins influence the localization of each GEF, but at this stage there are many gaps in our knowledge. For instance, new findings regarding DOCK8 clearly demonstrate important roles for this protein in immune cell polarization, but the molecular basis for its polarity is not yet known. In contrast, Tiam1 and *β*PIX have clear roles in T-cell polarity (particularly related to the immunological synapse) and are regulated by known interactions with members of the polarity network. Understanding how each of these players interact to dictate T-cell polarity will be the next big challenge.
